# Deliver CEBPE via cartilage targeting Lipid nanoparticle to block CEBPE-LTF-STAT3 positive feedback loop for efficient treatment of cartilage endplate degeneration

**DOI:** 10.1016/j.mtbio.2025.102027

**Published:** 2025-06-28

**Authors:** Jiangminghao Zhao, Peichuan Xu, Tao Li, Jinghong Yuan, Wenrui Zhao, Rui Ding, Guoyu Yang, Jiajun Xie, Guanfeng Huang, Zhidong Peng, Xinxin Miao, Xigao Cheng

**Affiliations:** a Department of Orthopaedics, The Second Affiliated Hospital, Jiangxi Medical College, Nanchang University, Nanchang, China; b Jiangxi Provincial Key Laboratory of Spine and Spinal Cord Disease, Nanchang University, Nanchang, China; c Institute of Minimally Invasive Orthopedics, Nanchang University, Nanchang, China

**Keywords:** Intervertebral disc degeneration, Lipid nanoparticle, Cartilage targeting, CEBPE, LTF, JAK2/STAT3 inflammatory signaling pathway

## Abstract

Intervertebral disc degeneration (IVDD) is recognized as a significant underlying factor contributing to clinical neck and low back pain. The cartilaginous endplates (CEP) serve as a selectively permeable barrier, which is essential for maintaining the internal homeostasis of the intervertebral disc (IVD). Dysfunction of the CEP is closely related to the initiation and progression of IVDD. In this paper, we demonstrate that CCAAT enhancer-binding protein *ε* (*CEBPE*) is crucial for the degeneration of the CEP. We observed a significant downregulation of *CEBPE* in CEP degeneration. The deficiency of *CEBPE* leads to inflammation, degradation of the extracellular matrix (ECM), and calcification of endplate chondrocytes (EPCs). Conversely, overexpression of *CEBPE* mitigates these detrimental processes. Mechanistically, *CEBPE* deficiency down-regulates the transcription level of lactoferrin (*LTF*), which in turn activates the JAK2/STAT3 inflammatory signaling pathway, and STAT3 inhibits the transcription of *CEBPE*. These findings unveil a previously unidentified positive feedback loop, CEBPE-LTF-STAT3, that modulates EPCs degeneration. Importantly, a lipid nanoparticle targeting chondrocytes for the efficient delivery of a *CEBPE*-overexpressing plasmid significantly reduced ECM degradation and the calcification process in CEP, substantially attenuating the progression of IVDD. This highlights promising targets and effective strategies for mitigating the impact of IVDD.

## Graphical abstract

A positive feedback loop involving CEBPE-LTF-STAT3 was identified in cartilage endplate degeneration and a lipid nanoparticle with cartilage-targeting capabilities was innovatively developed to deliver the *CEBPE* overexpression plasmid to the cartilage endplate site, thereby effectively treating cartilage endplate degeneration.

**Figure:**
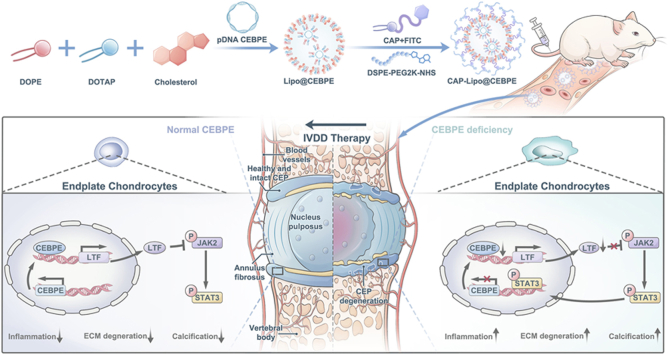

## 1 Introduction

Neck and low back pain are prevalent clinical conditions and rank among the leading global causes of disability [1,2]. Among the various etiologies contributing to these symptoms, intervertebral disc degeneration (IVDD) is recognized as a major underlying factor [3,4]. IVDD is characterized by intricate pathophysiological processes, encompassing disc inflammation, imbalances in extracellular matrix (ECM) synthesis and degradation, and dysregulation of cellular signaling pathways [5]. Despite extensive research, the precise mechanisms driving IVDD pathogenesis remain elusive. At present, no curative treatments exist for IVDD; surgical intervention is often reserved for symptom relief when conservative approaches fail [6]. This underscores the critical need for comprehensive studies into the molecular mechanisms governing IVDD initiation and progression to facilitate the development of innovative biological therapies targeting the condition.

The intervertebral disc (IVD), situated between adjacent vertebral bodies, is composed of three distinct components: the nucleus pulposus (NP), annulus fibrosus (AF), and cartilaginous endplates (CEP). It provides spinal stability and flexibility while serving the critical function of absorbing and transmitting mechanical loads [7]. Traditionally, research has predominantly focused on pathological changes within the NP and AF. However, it is crucial to emphasize that the IVD, the largest avascular tissue in the human body, relies extensively on the CEP for nutrient supply. The CEP acts as a selectively permeable barrier, enabling the exchange of nutrients, metabolites, water, and ions between the IVD and the adjacent vertebral bodies, which is essential for maintaining the internal homeostasis of the IVD [[8], [9], [10], [11]]. Evidence suggests that ECM degradation or calcification within the CEP markedly hinders nutrient diffusion, thereby contributing significantly to the progression of IVDD [12,13]. Despite its critical role, research on the CEP remains limited, and the mechanisms underlying CEP dysfunction in IVDD pathogenesis are largely unexplored. This highlights an urgent need to investigate the specific pathways involved in CEP degeneration during IVDD progression.

CCAAT enhancer-binding protein *ε* (*CEBPE*) is a 31-kDa protein classified as a member of the basic leucine zipper (bZIP) transcription factor family [14]. The C/EBP family is integral to the growth and differentiation of monocytes, macrophages, granulocytes, T cells, and related cell types. Additionally, it regulates the expression of several inflammation-associated cytokines, including TNF, IL-1β, and IL-12, etc [[15], [16], [17]]. Inflammation plays a crucial role in endplate cartilage remodeling and calcification, exhibiting a positive correlation with degeneration. During inflammatory states, pro-inflammatory factors induce the upregulation of bone morphogenetic protein and calcium-sensing receptor transcription factor proteins, thereby accelerating the remodeling and calcification of endplate cartilage and ultimately contributing to IVDD [18,19]. Despite its importance, the precise role of *CEBPE* in the pathogenesis of IVDD remains unclear.

In this study, we present a significant finding that during the progression of IVDD, *CEBPE* deficiency triggers a positive feedback loop CEBPE-LTF-STAT3, promoting inflammatory responses, ECM degradation and calcification of endplate chondrocytes (EPCs). Mechanically, CEBPE directly interacts with the promoter sequence of lactoferrin (*LTF*), and its downregulation limits LTF expression, subsequently activating the JAK2/STAT3 signaling pathway. Additionally, STAT3 also acts as a transcriptional regulator of *CEBPE*, its upregulation suppresses CEBPE expression. Since the U.S. Food and Drug Administration approved solid lipid nanoparticles (LNPs) in 2018, LNPs have rapidly become the preferred drug delivery platform in the biopharmaceutical industry [20]. An innovative CAP-Lipo@CEBPE system was developed, which integrates cartilage affinity peptide (CAP) with lipid nanoparticles to encapsulate the *CEBPE* plasmid and deliver it to the CEP via the peripheral circulation. This system substantially attenuated the progression of IVDD, indicating promising targets and efficient strategies for ameliorating the impact of IVDD.

## 2 Methods

### 2.1 Ethics declaration

Approval for this study was granted by the Ethics Committee of the Second Affiliated Hospital of Nanchang University (Approval No. Review (2023) No. (06)). Tissue samples from cervical CEP were obtained from patients undergoing discectomy at the hospital. All participants provided written informed consent for the utilization of their tissue specimens in research.

### 2.2 Clinical samples

CEP tissue samples were categorized into two groups based on Pfirrmann grading: Control group (grades I–II, n = 15): Samples sourced from patients undergoing decompression surgery for neurological dysfunction due to cervical spine trauma. Degenerative (Deg) group (grades III–V, n = 15): Samples collected from patients with degenerative cervical myelopathy undergoing discectomy. Preoperative magnetic resonance imaging (MRI) was performed for all patients in accordance with established protocols [21], enabling the classification of the endplate tissues based on imaging findings. The CEP tissues collected during surgery were promptly frozen in liquid nitrogen and stored at −80 °C for further analysis. Detailed patient information and sample specifications are provided in Supplementary Table 1.

### 2.3 Histochemical, immunohistochemical (IHC), and immunofluorescence (IF) techniques

Human CEP tissues and rat tail IVD tissues were fixed in 4 % paraformaldehyde (PFA; Biosharp, BL539A). The tissues were embedded in paraffin and subsequently sectioned. Paraffin-embedded sections underwent hematoxylin and eosin (H&E) staining, SafraninO staining, IHC and IF analyses. For IHC and IF staining, sections were incubated with the following primary antibodies: CEBPE (1:100, Santa Cruz, sc-515192), Collagen II (1:800, Proteintech, 28459-1-AP), MMP13 (1:100, Proteintech, 18165-1-AP), RUNX2 (1:500, Servicebio, GB115631), OPN (1:500, Servicebio, GB112328-100), phosphorylated JAK2 (p-JAK2) (1:800, Abcam, ab32101), and phosphorylated STAT3 (p-STAT3) (1:800, Abcam, ab267373). After primary antibody incubation, sections were co-incubated with the corresponding secondary antibodies at 37 °C for 1 h. IHC staining was visualized using a DAB substrate kit (Servicebio, G1212) and counterstained with hematoxylin (Servicebio, G1040). IF-stained sections were mounted using an antifade mounting medium (Servicebio, G1401) and examined under a bright-field microscope (Nikon Eclipse E100, Japan) and a fluorescence microscope (Nikon Eclipse C1, Japan). Image analysis was conducted using ImageJ software.

In parallel, fresh tissues were lightly blotted with filter paper or gauze to remove surface moisture, rapidly frozen in liquid nitrogen for 15 s, and stored at −80 °C. These tissues were later embedded in an OCT compound to prepare frozen sections. After equilibration to room temperature, frozen sections were stained with DAPI and examined under a fluorescence microscope (Nikon Eclipse C1, Japan).

### 2.4 Cell cultivation and cell transfection

The human endplate chondrocyte cell line was cultured at 37 °C in a humidified atmosphere containing 5 % CO_2_. The cells were maintained in Dulbecco's Modified Eagle Medium/Nutrient Mixture F-12 (DMEM/F12; ThermoFisher) supplemented with 10 % fetal bovine serum (FBS; ThermoFisher) and 1 % penicillin-streptomycin (Invitrogen). The cells were grown in 12-well plates. When cell confluence reached 60–80 %, transfection was performed using riboFECT™ CP transfection reagent (RiboBio, R10035.5) or Lipofectamine 3000 (ThermoFisher, L3000008), following the manufacturer's protocols.

### 2.5 Real-time quantitative polymerase chain reaction (RT-qPCR)

Total RNA was extracted from CEP tissues or cultured cells using TRIzol reagent (ThermoFisher, Cat. No: 15596026CN). RNA purity and concentration were determined with a NanoDrop One spectrophotometer (ThermoFisher, USA). To specifically analyze mRNA expression, the RNA samples were treated with RNase R to remove linear transcripts before reverse transcription into cDNA using the PrimeScript RT reagent kit (TaKaRa, Cat. No: RR037A). Quantitative PCR was performed using the ABI 7500 real-time PCR system (ThermoFisher, USA) with TB Green Premix Ex Taq II reagent (TaKaRa, Cat. No: RR820A). The 2^−^ΔΔCt method was employed to analyze relative gene expression. All RT-qPCR primers were synthesized by RiboBio (Guangzhou, China), and detailed primer sequences are provided in Supplementary Table 2.

### 2.6 Western blot (WB)

CEP tissues and cells were lysed on ice for 30 min using a lysis buffer (Beyotime, Cat. No: P0013). The lysates were mixed with 6 × concentrated loading buffer in a 1:5 ratio and heated at 100 °C for 15 min to denature proteins. Denatured proteins were separated using SDS-PAGE and transferred onto PVDF membranes (Millipore, USA). The membranes were blocked with QuickBlock™ Western blocking buffer (Beyotime, Cat. No: P0252) at room temperature for 30 min, followed by overnight incubation at 4 °C with the following primary antibodies: CEBPE (1:1000, Santa Cruz, sc-515192), Collagen II (1:1000, Proteintech, 28459-1-AP), MMP13 (1:1000, Proteintech, 18165-1-AP), RUNX2 (1:1000, Abcam, ab236639), OPN (1:1000, Abcam, ab166709), IL-1β (1:1000, Proteintech, 26048-1-AP), TNF (1:1000, Proteintech, 17590-1-AP), JAK2 (1:1000, Abcam, ab108596), P-JAK2 (1:1000, Abcam, ab32101), STAT3 (1:1000, Abcam, ab68153), P-STAT3 (1:1000, Abcam, ab267373), α-Tubulin (1:10000, Proteintech, 11224-1-AP), and GAPDH (1:50000, Proteintech, 60004-1-Ig). Following incubation, the membranes underwent three washes with TBST buffer, then were incubated with the respective secondary antibodies at ambient temperature for a duration of 2 h. Detection of chemiluminescence was accomplished using an advanced chemiluminescent reagent (UElandy, Product Code: S6009M), and the resultant signals were captured using the Bio-Rad ChemiDoc Touch imaging system.

### 2.7 Enzyme-linked immunosorbent assay (ELISA)

Commercial ELISA kits were used to measure the concentrations of IL-1β (Thermo Fisher, Cat. No: 88–7261) and TNF (Servicebio, Cat. No: GEH0004) in the supernatants.

### 2.8 Von Kossa staining

Cell calcification was assessed utilizing the Von Kossa Staining Kit from Solarbio (Cat. No: G3282). Upon reaching 60–80 % confluence in 12-well plates, the cells were washed twice with phosphate-buffered saline (PBS) and subsequently fixed with 4 % PFA for 15 min. After fixation, the cells were rinsed with distilled deionized water. They were then treated with Von Kossa silver nitrate solution and exposed to UV light for 1 h, followed by two rinses with distilled water. Subsequently, the cells were incubated in a reducing agent (Hypo solution) for 5 min in darkness. The cells were then stained with hematoxylin for 2 min and counterstained with eosin for 1 min. After thorough washing and drying, the samples were examined under a Nikon Eclipse E100 microscope (Nikon, Japan), where calcium deposits appeared as dark black structures.

### 2.9 Chromatin immunoprecipitation (ChIP) assays and ChIP-seq

The cells were crosslinked using formaldehyde with a final concentration of 1 %, followed by DNA lysis and sonication. The immunoprecipitation (IP) was conducted overnight at 4 °C. Antibodies used for ChIP analysis included anti-CEBPE (Santa Cruz, sc-515192), anti-IgG, and anti-RNA Pol II. Protein A/G magnetic beads were added to each IP sample and incubated at 4 °C for 4 h. After IP elution, DNA was recovered for subsequent ChIP-PCR or ChIP-Seq analysis.

The ChIP-Seq library was prepared and sequenced by Aksomics (Shanghai) Biotechnology Co., Ltd. Sequencing was carried out on the Illumina NovaSeq 6000 system, utilizing the NovaSeq 6000 S4 reagent kit (300 cycles), in accordance with the manufacturer's guidelines. Image analysis and base calling were conducted using the Off-Line Basecaller software (OLB, version 1.8). Subsequently, the quality of the sequences was evaluated with FastQC software. Clean reads, which were obtained following filtering with the Solexa CHASTITY quality control filter, were mapped to the human reference genome (UCSC HG19) using BOWTIE2version 2.2.7.

### 2.10 ChIP-PCR

The cells were incubated with either the anti-CEBPE antibody (Santa Cruz, sc-515192) or anti-p-STAT3 antibody (Abcam, ab267373), and the mixture was rotated at 4 °C overnight to ensure thorough mixing. The following day, Protein G magnetic beads were added to capture the antibody-bound protein-DNA complexes, which were subsequently eluted. The crosslinks between proteins and DNA were then reversed. The resulting DNA was subjected to proteinase K digestion followed by purification. The purified DNA templates were used in real-time PCR to quantify the amount of DNA immunoprecipitated by the specific antibodies.

### 2.11 Luciferase reporter assay

Wild-type and mutant plasmids containing the *LTF* and *CEBPE* promoters were constructed by Focus Biosciences Co., Ltd. To analyze the activity of the *LTF* or *CEBPE* promoter, Lipofectamine 2000 (ThermoFisher, 11668019) was used to transfect cells in a 6-well plate with DNA containing the *LTF* or *CEBPE* promoters, along with *CEBPE* or *STAT3* plasmids. 36 h post-transfection, the Dual-Luciferase Reporter Assay Kit (GeneCreate, JKR23008) was used to measure the activities of firefly and Renilla luciferases.

### 2.12 Preparation of CAP-Lipo@CEBPE

The ultrasonic dispersion method was used to prepare cationic liposomes, as described in previous studies [22,23]. Briefly, DOTAP, DOPE, and cholesterol (Macklin, China) were mixed in chloroform and methanol to create a 25 mg/mL solution. The mixture was evaporated in a rotary evaporator at 40 °C. After removing the rotary evaporation flask, PBS was introduced into the mixture, which was then sonicated. Uniform-sized nanoliposomes were subsequently obtained by passing the mixture through a filter sourced from Millipore (Germany). The liposomes' morphology was inspected under a transmission electron microscope (TEM). Furthermore, their size, zeta potential, and polydispersity index (PDI) were determined by dynamic light scattering (DLS) employing a Zetasizer(Malvern Zetasizer Nano ZS90, UK).

Subsequently, DSPE-PEG2000-NHS was dissolved in DMF. K(FITC)DWRVIIPRPSA (FITC-CAP) (Apeptide Co., Ltd), and triethylamine were added to achieve complete dissolution. After the reaction at room temperature, the mixture was dialyzed against pure water for 24 h. The solution was then collected and dialyzed, followed by freeze-drying and molecular sieve purification to obtain DSPE-PEG2000-FITC-CAP. The chemical structure of the final product was confirmed using nuclear magnetic resonance (NMR) spectroscopy (Supplementary Fig. 1). Subsequently, DSPE-PEG2000-FITC-CAP and cationic liposomes were dissolved in a 4 % ethanol solution, incubated, and thoroughly mixed to yield the desired liposomes (CAP-Lipo). To prepare DNA-encapsulated lipid nanoparticles, CAP-Lipo and plasmid DNA were each diluted with 5 % glucose. The diluted CAP-Lipo (8 mM) and DNA (500 μg/mL) were then mixed at a 1:1 M ratio and incubated at room temperature for 30 min to generate CAP-Lipo@CEBPE.

### 2.13 Animal model

Eight-week-old male Sprague-Dawley rats, weighing between 200 and 250 g (n = 20), were acquired from Changsha Tianqin Biotechnology Co., Ltd. The rats were subsequently randomized into four groups: a control group, an IVDD group, an IVDD + Lipo@CEBPE group, and an IVDD + CAP-Lipo@CEBPE group. The rats in the IVDD group, IVDD + Lipo@CEBPE group, and IVDD + CAP-Lipo@CEBPE group underwent general anesthesia induced by 2 % isoflurane, followed by the insertion of a 27 G needle through the tail skin into the IVD. The needle was left in positon for 1 min [24]. One week after modeling, the rats in the IVDD + Lipo@CEBPE group and IVDD + CAP-Lipo@CEBPE group received intravenous injections of either Lipo@CEBPE or CAP-Lipo@CEBPE (1.1 × 10^10^ particles/1 mL/rat), administered once every four days and continued to inject for one month. Unlike retroviral vector-based gene therapy/transduction with stable expression of transgenes, lipid nanoparticle/plasmid-based gene therapy/transfection results in transient gene expression [25]. All experimental protocols were approved by the Animal Ethics Committee at Nanchang University (Approval No.NCULAE-20221031075).

### 2.14 Radiological examination

After induction of general anesthesia with 2 % isoflurane, X-ray imaging was performed. And the Disc Height Index (DHI) was calculated using the methodology described in a previous publication [26] as an indicator of IVDD severity. Additionally, an MRI was conducted on the rat tails. All subjects underwent sagittal T2-weighted imaging using a 4.7T permanent magnet MRI scanner. The resulting MRI images were evaluated in a blinded manner according to the IVDD classification framework [26].

### 2.15 Micro-computed tomography (μCT) analysis

Following the euthanasia of the rats, in order to maintain the tissue structure, tail tissue samples were immediately fixed with 4 % PFA, then tail tissue samples were dissected and subjected to μCT imaging using the NEMO NMC-200 scanner. The obtained images were reconstructed with NRecon v1.7 software. For image visualization and analysis, the Cruiser and Avatar software packages were utilized.

### 2.16 Statistical analysis

Data are presented as mean ± standard deviation. Statistical comparisons between the two groups were performed using an unpaired two-tailed Student's t-test. For comparisons among multiple groups, one-way or two-way analysis of variance (ANOVA) was applied, followed by post hoc Tukey's test. Statistical significance was defined as ∗P < 0.05, ∗∗P < 0.01, and ∗∗∗P < 0.001. All analyses were conducted using GraphPad Prism 10.1.2.

## 3 Results

### 3.1 *CEBPE* expression is decreased in degenerative CEP

Patients were categorized into Control and Deg groups according to their preoperative MRI Pfirrmann grade classifications (Fig. 1A). CEP tissue samples were harvested and analyzed using H&E and Safranin O staining to evaluate structural integrity and cellular composition in the Control and Deg groups (Fig. 1B and C). Prior research from our group [27] provided a detailed characterization of mRNA expression profiles in control and degenerative CEP samples through hierarchical clustering, displaying the top 20 differentially expressed genes (DEGs) (Fig. 1D). To investigate essential transcript factors (TFs) in the degenerative CEP, we integrated the Transcription factor database TRRUST (http://www.grnpedia.org/trrust), JASPAR database (https://jaspar.genereg.net/) and TOP20 DEGs, *CEBPE* were the only TF screened (Fig. 1E). Compared to Control group, 246 DEGs were found to be differentially regulated, *CEBPE* is downregulated in the Deg group (Fig. 1F). The complete microarray dataset is publicly available in the Gene Expression Omnibus (GEO) database under accession number GSE153761. A series of molecular and histological analyses were performed, including RT-qPCR, WB, IF, and IHC staining. These experiments revealed a pronounced downregulation of CEBPE expression in degenerative CEP tissues (Fig. 1G–J). Collectively, these findings suggest a potential role for *CEBPE* in the pathological mechanisms underlying CEP degeneration.

**Fig. 1:**
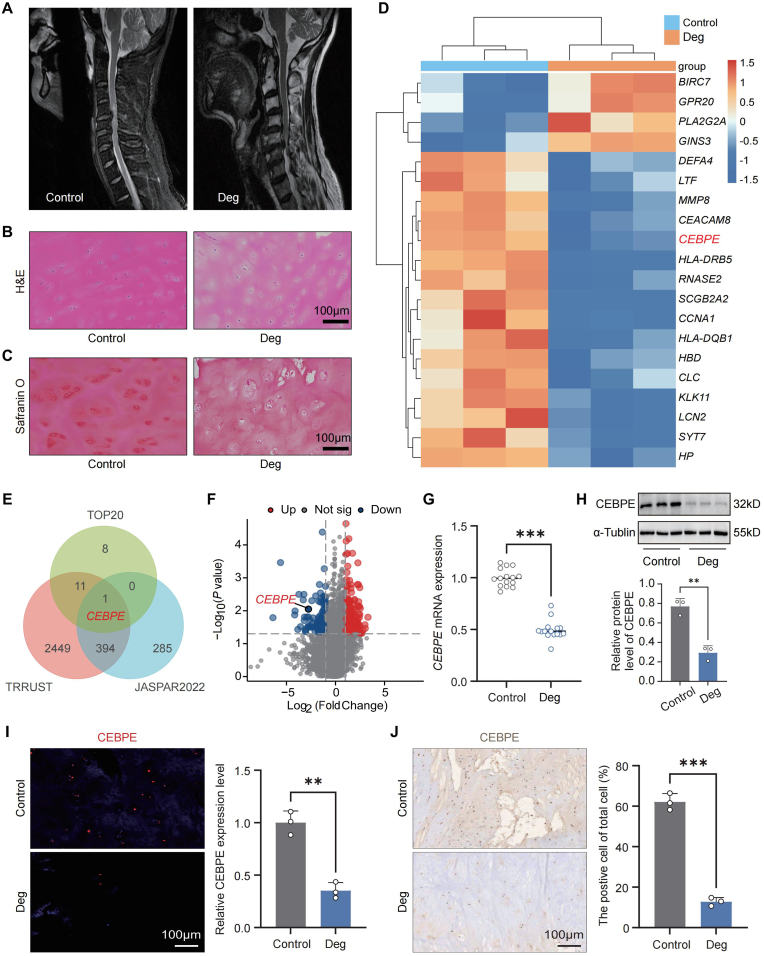
***CEBPE* expression is decreased in degenerative CEP.** (A) Preoperative MRI results of patients in the Control group (Pfirrmann grades I-II) and Deg group (Pfirrmann grades III -V). (B) H&E staining of CEP in the Control group and Deg group (Scale Bar = 100 μm). (C) Safranin O staining of CEP in the Control group and Deg group (Scale Bar = 100 μm). (D) Heat map of differentially expressed TOP20 mRNA in the Control group and Deg group (fold change≥2; p < 0.05). (E) Venn diagram showing overlapping TFs involved in the top 20 DEGs, TRRUST, and JASPAR database. (F) Volcano plot showing DEGs in CEP tissues from Control and Deg group. (G) RT-qPCR analysis of the relative levels of *CEBPE* mRNA transcripts in the Control group (n = 15) and Deg group (n = 15). (H) WB analysis of CEBPE expression in the Control group and Deg group (n = 3). (I) IF staining and quantification of CEBPE expression in the Control group and Deg group (n = 3). (J) IHC staining and quantification of CEBPE expression in the Control group and Deg group (n = 3). ∗∗P < 0.01, and ∗∗∗P < 0.001.

### 3.2 *CEBPE* deficiency induces inflammatory responses, ECM degradation and calcification in EPCs

To investigate the functional role of *CEBPE* in CEP degeneration, Given the critical role of the pro-inflammatory cytokine IL-1β in IVDD progression [4], EPCs were exposed to IL-1β at various concentrations and durations. WB and IF analyses revealed a significant reduction in CEBPE expression in IL-1β-treated EPCs (Fig. 2A and B). To further delineate the effects of *CEBPE* on EPCs, small interfering RNA (siRNA) was employed to silence *CEBPE* expression. The results showed that the expression of *CEBPE* was significantly inhibited (Fig. 2C and D). The ELISA results indicated that the concentrations of IL-1β and TNF, which are the two most important cytokines in IVDD [4], were notably elevated in the Si-CEBPE group compared to the Si-NC group (Fig. 2E). The comparable trend of mRNA transcripts for *IL-1β* and *TNF* was observed in EPCs by RT-qPCR (Fig. 2F). Similar to the efficacy of EPCs regulating inflammatory cytokines at the transcriptional level, WB also confirmed that IL-1β and TNF

**Fig. 2:**
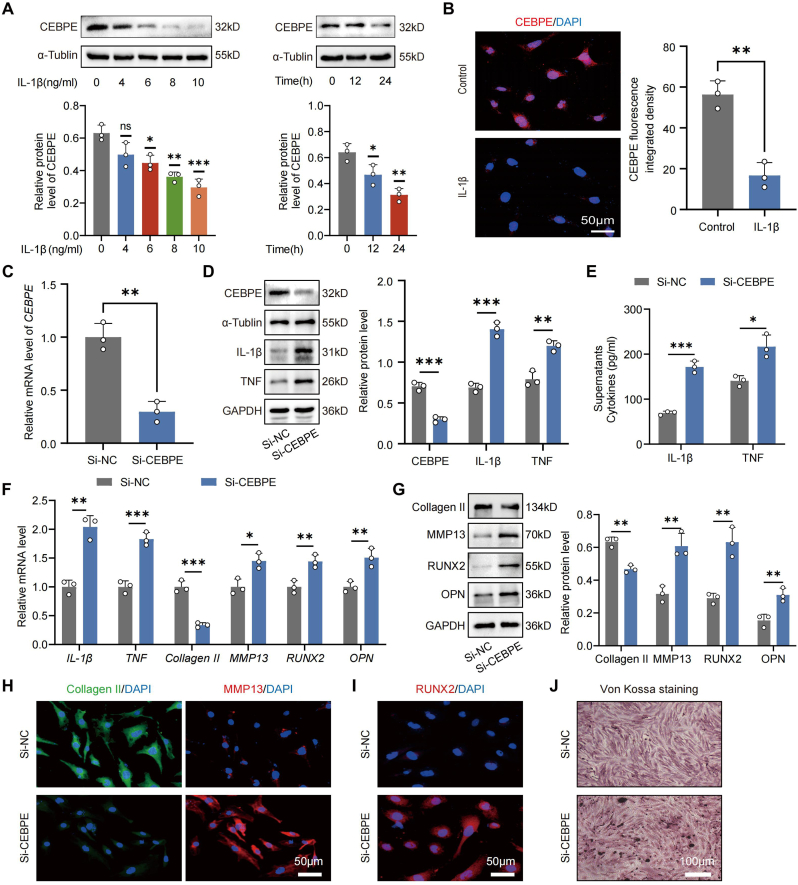
***CEBPE* deficiency induces Inflammatory Responses, ECM degradation and calcification in EPCs.** (A) WB analysis of CEBPE expression in EPCs that had been treated with the indicated doses and time of IL-1β (n = 3). (B) IF staining and quantification of CEBPE expression in EPCs treated with, or without, IL-1β (10 ng/mL) (n = 3) (Scale bar = 50 μm). (C) RT-qPCR analysis of the relative levels of *CEBPE* mRNA transcripts in EPCs treated with Si-NC, or with Si-CEBPE (n = 3). (D) WB analysis of CEBPE, IL-1β TNF expression in EPCs treated with Si-NC, or with Si-CEBPE (n = 3). (E) ELISA quantification of the levels of IL-1β and TNF in the supernatants of EPCs treated with Si-NC, or with Si-CEBPE (n = 3). (F) RT-qPCR analysis of the relative levels of *Collagen II*, *MMP13*, *RUNX2*, *OPN*, *IL-1β*, and *TNF* mRNA transcripts in EPCs treated with Si-NC, or with Si-CEBPE (n = 3). (G) WB analysis of Collagen II, MMP13, RUNX2, OPN expression in EPCs treated with Si-NC, or with Si-CEBPE (n = 3). (H, I) Representative IF staining of Collagen II, MMP13, and RUNX2 in EPCs treated with Si-NC, or with Si-CEBPE (Scale bar = 50 μm). (J) Representative Von Kossa staining in EPCs treated with Si-NC, or with Si-CEBPE (Scale bar = 100 μm). ∗P < 0.05, ∗∗P < 0.01, and ∗∗∗P < 0.001.

expression in EPCs, these inflammation-related proteins were clearly higher in the Si-CEBPE group than those in the Si-NC group (Fig. 2D). Wike et al. have reported that the molecular mechanism CEP degeneration involved the up-regulation of matrix-degrading enzymes and inflammatory cytokines [28]. The negative effects of *CEBPE* deficiency on Collagen II and MMP13 expression in EPCs were observed by WB and IF staining (Fig. 2H and I). CEP degeneration is characterized by the loss of ECM and the calcification of hyaline cartilage [29]. We investigated CEP calcification in vitro, its effects on the expression of osteogenic markers *RUNX2* and *OPN* were examined by RT-qPCR. The mRNA expression of *RUNX2* and *OPN* were clearly higher in the Si-CEBPE group than those in the Si-NC group (Fig. 2F). Similar to the efficacy of EPCs regulating *RUNX2* and *OPN* at the transcriptional level, RUNX2 and OPN protein expression increased in the Si-CEBPE group (Fig. 2G). IF staining was used to further confirm the changes in RUNX2 protein expression levels in response to *CEBPE* deficiency (Fig. 2I). Von Kossa staining also revealed that *CEBPE* deficiency promoted calcium nodule formation in EPCs (Fig. 2J). These observations underscore the role of *CEBPE* deficiency in promoting inflammatory responses, ECM degradation and calcification in EPCs.

### 3.3 *CEBPE* overexpression alleviated IL-1β-induced the ECM degradation and calcification in EPCs

To further elucidate the role of *CEBPE* on IL-1β-induced ECM degradation and calcification in EPCs, we tested the effect of *CEBPE* overexpression on the expression of *Collagen II*, *MMP13*, *RUNX2* and *OPN*. While treatment with IL-1β increased the relative levels of *Collagen II*, *MMP13*, *RUNX2* and *OPN* expression in EPCs, Plasmids to enhance *CEBPE* could reverse its effect on transcriptional level (Fig. 3A). In addition, the result of WB analysis was basically consistent with RT-qPCR analysis (Fig. 3B). Notably, IF staining of Collagen II, MMP13 and RUNX2 further confirmed the effect of *CEBPE* (Fig. 3C and D). Von Kossa staining also revealed that IL-1β-induced calcium nodule formation in EPCs, *CEBPE* overexpression decreased calcium nodule (Fig. 3E). Overall, these results highlight *CEBPE* as a critical regulatory factor in EPCs, playing a pivotal role in modulating inflammatory responses and protecting against chondrocyte degeneration.

**Fig. 3:**
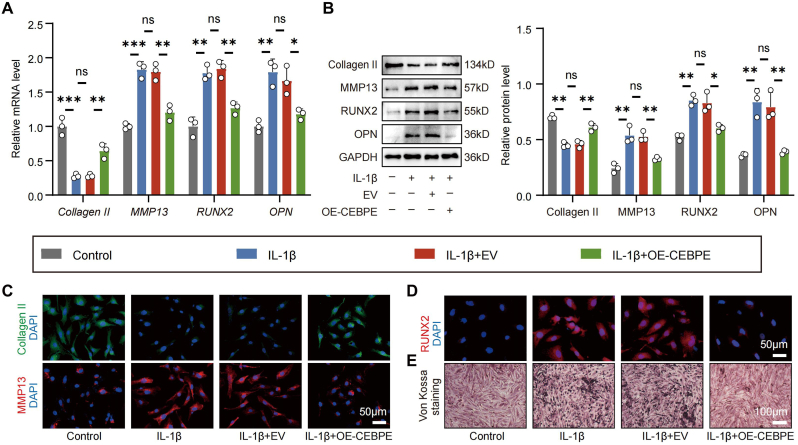
***CEBPE* overexpression alleviated IL-1β-induced the ECM degradation and calcification in EPCs.** (A) RT-qPCR analysis of the relative levels of *Collagen II*, *MMP13*, *RUNX2* and *OPN* mRNA transcripts in OE-CEBPE or Empty Vector (EV) EPCs that had been pretreated with or without IL-1β (n = 3). (B) WB analysis of Collagen II, MMP13, RUNX2, and OPN expression in OE-CEBPE or EV EPCs that had been pretreated with or without IL-1β (n = 3). (C, D) Representative IF staining of Collagen II, MMP13, and RUNX2 in OE-CEBPE or EV EPCs that had been pretreated with or without IL-1β (Scale bar = 50 μm). (E) Representative Von Kossa staining in OE-CEBPE or EV EPCs that had been pretreated with or without IL-1β (Scale bar = 100 μm). n.s. not significant, ∗P < 0.05, ∗∗P < 0.01, and ∗∗∗P < 0.001.

### 3.4 CEBPE bound to the *LTF* promoter to regulate LTF expression in EPCs

Previous studies have established that *CEBPE* functions as a transcription factor involved in cellular developmental processes [[15], [16], [17]]. To explore its function in EPCs, we conducted a comprehensive genome-wide ChIP-seq analysis (Supplementary Fig. 2A). By integrating ChIP-seq data with the TOP20 mRNA expression profile of human cartilage endplate tissues (GSE153761) (Supplementary Fig. 2B) and the TRRUST database (Supplementary Fig. 2C), *LTF* was identified as a key target gene potentially regulated by CEBPE in EPCs (Fig. 4A). IF assays revealed cell localization of the CEBPE and LTF (Fig. 4B). We delved deeper into the regulatory interplay between *CEBPE* and *LTF*. Specifically, *LTF* mRNA levels exhibited a notable decrease in EPCs transfected with Si-CEBPE compared to those treated with Si-NC (Fig. 4C). Similar to effects of *CEBPE* deficiency on LTF protein expression in EPCs were observed by WB and IF staining (Fig. 4D and E). A detailed analysis of ChIP-seq data identified significant peak regions near the *LTF* promoter sequence (Fig. 4F).

**Fig. 4:**
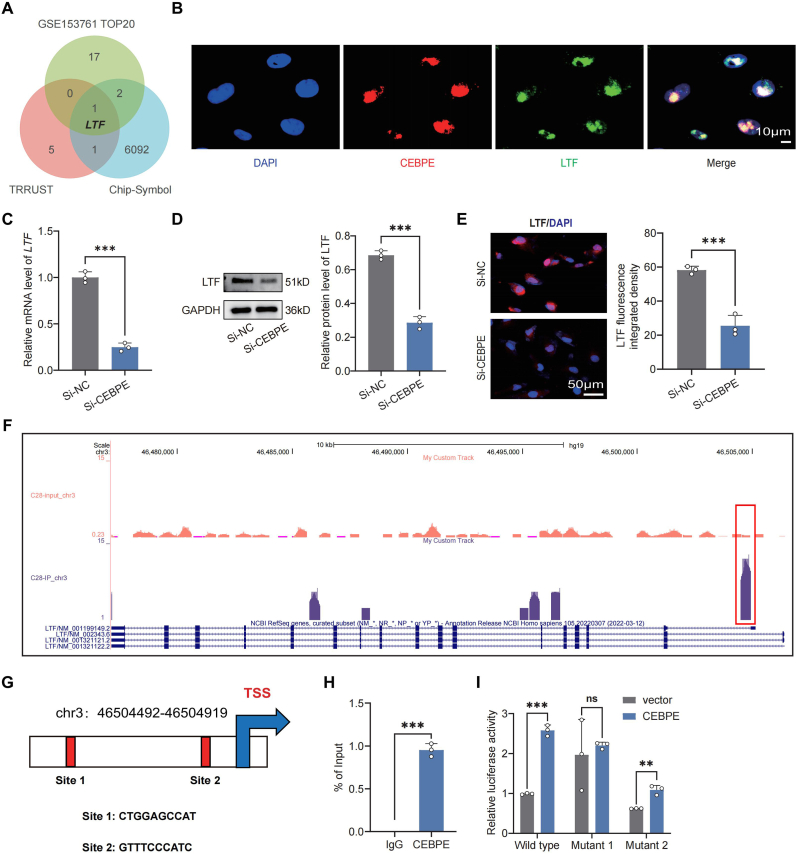
**CEBPE bound to the *LTF* promoter to regulate LTF expression in EPCs.** (A) Venn diagram showing overlapping relevant target genes of CEBPE involved in the ChIP-seq, TOP20 DEGs in GSE153761, and TRRUST. (B) Immunofluorescent localization of CEBPE and LTF in EPCs (Scale bar = 10 μm). (C) RT-qPCR analysis of the relative levels of *LTF* mRNA transcripts in EPCs treated with Si-NC, or with Si-CEBPE (n = 3). (D) WB analysis of LTF expression in EPCs treated with Si-NC, or with Si-CEBPE (n = 3). (E) IF staining and quantification of CEBPE expression in EPCs treated with Si-NC, or with Si-CEBPE (n = 3). (F) The prominent peak regions of *LTF* promoter sequence. (G) Schematic diagram of two potential CEBPE binding sites (Site 1 and Site 2) in the *LTF* promoter. (H) ChIP-PCR for CEBPE enrichment in the *LTF* promoter. (I) The binding relationship between CEBPE and *LTF*-promoter as examined by dual-luciferase reporter gene assay. ∗∗P < 0.01, and ∗∗∗P < 0.001.

Using the JASPAR database, two putative transcription factor binding sites within the *LTF* promoter were predicted for experimental validation (Fig. 4G). ChIP-PCR results showed that *LTF* enrichment in CEBPE in the presence of CEBPE was notably higher than that in the IgG control sample (Fig. 4H). Furthermore, dual-luciferase reporter assays were performed to validate the predicted CEBPE binding site within the *LTF* promoter. The outcomes of these assays revealed that mutating the first binding site (Mutant1) completely abolished luciferase activity, thereby confirming its essential function (Fig. 4J). Collectively, these observations imply that CEBPE directly modulates *LTF* transcription in chondrocytes through binding to specific regions of the *LTF* promoter.

### 3.5 The feedback loop CEBPE-LTF-STAT3 in the ECM degradation and calcification of EPCs

An investigation was undertaken to determine whether *CEBPE* exerts its modulatory effects on the degeneration of EPCs through the regulation of *LTF* expression. The ELISA results indicated that *CEBPE* silencing notably elevated the concentrations of IL-1β and TNF, which were reduced by transfection with *LTF*-overexpressing plasmids (Fig. 5B). Consistent trends were observed in RT-qPCR and WB analyses, where both transcriptional and protein expression levels of the inflammatory cytokines IL-1β and TNF were diminished (Fig. 5C and D). In addition, *CEBPE* silencing enhanced the *MMP13*,*RUNX2* and *OPN* mRNA expression, but reduced *Collagen II* mRNA expression in EPCs, which were mitigated or abrogated by *LTF* overexpression (Fig. 5C). In parallel, WB and IF assays revealed the same changing trend with transcriptional level (Fig. 5E–G). And transfection with *LTF*-overexpressing plasmids in Si-CEBPE EPCs dramatically decreased the calcium nodules induced by *CEBPE* silencing (Fig. 5H). These results collectively indicate that *CEBPE* modulates chondrocyte degeneration by regulating *LTF* expression, implicating *LTF* as a critical mediator in this upstream regulatory mechanism.

**Fig. 5:**
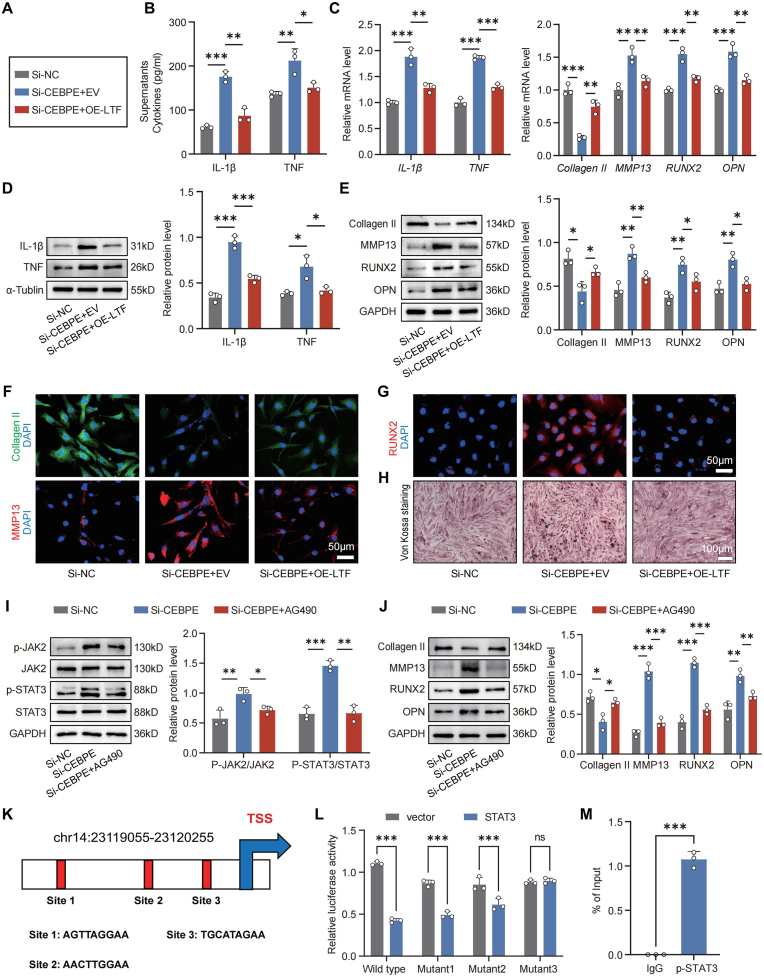
**The feedback loop between CEBPE and the LTF/JAK2/STAT3 pathway in the ECM degradation and calcification of EPCs.** (A) Experimental grouping diagram. (B) ELISA quantification of the levels of IL-1β and TNF in the supernatants of Si-NC or Si-CEBPE EPCs that had been transduced with *LTF*-overexpressing plasmids (OE-LTF), or with EV (n = 3). (C) RT-qPCR analysis of the relative levels of *IL-1β*, *TNF*, *Collagen II*, *MMP13*, *RUNX2*, and *OPN* mRNA transcripts in Si-NC or Si-CEBPE EPCs that had been transduced with OE-LTF, or with EV (n = 3). (D, E) WB analysis of IL-1β, TNF, Collagen II, MMP13, RUNX2, and OPN expression in Si-NC or Si-CEBPE EPCs that had been transduced with OE-LTF, or with EV (n = 3). (F, G) Representative IF staining of Collagen II, MMP13, and RUNX2 in Si-NC or Si-CEBPE EPCs that had been transduced with OE-LTF, or with EV. (H) Representative Von Kossa staining in Si-NC or Si-CEBPE EPCs that had been transduced with Si-LTF, or with EV. (I, J) WB analysis of Collagen II, MMP13, RUNX2 and OPN expression in Si-NC or Si-CEBPE EPCs that had been treated with or without AG490 (n = 3). (K) Schematic diagram of three potential STAT3 binding sites (Site 1, Site 2 and Site 3) in the *CEBPE* promoter. (L) The binding relationship between STAT3 and *CEBPE*-promoter as examined by dual-luciferase reporter gene assay. (M) ChIP-PCR for p-STAT3 enrichment in the *CEBPE* promoter. n.s. not significant, ∗P < 0.05, ∗∗P < 0.01, and ∗∗∗P < 0.001.

Our previous research [30] revealed that *LTF* ameliorates cartilage endplate degeneration by suppressing ECM degradation and calcification through the JAK2/STAT3 pathway. This study established that CEBPE functions as a transcriptional regulator of *LTF*. Thus, the JAK2/STAT3 signaling pathway emerges as a pivotal mechanism in the effects of *CEBPE* deficiency on CEP degeneration. WB analysis demonstrated that knockdown of *CEBPE* augmented JAK2/STAT3 signaling, along with increased expression of MMP13, RUNX2, and OPN, while concurrently decreasing Collagen II levels in EPCs. These alterations were alleviated by administration of the JAK2/STAT3 pathway inhibitor AG490 (Fig. 5I and J). Interestingly, analysis using the UCSC Genome Browser (https://genome.ucsc.edu/) revealed that STAT3, acting as a transcription factor, is located within the promoter region of *CEBPE* (Supplementary Fig. 3A). And after overexpression of *STAT3* in EPCs, we observed a significant suppression in the mRNA and protein expression of *CEBPE* (Supplementary Fig. 3B and C). Using the JASPAR database, the potential binding sites of STAT3 on the *CEBPE* promoter were predicted (Fig. 5K). Based on these predictions, a dual-luciferase reporter assay was performed on the top three binding site sequences, confirming that luciferase activity was abolished in Mutant3 (Fig. 5L). Furthermore, ChIP-PCR data showed that *CEBPE* enrichment in p-STAT3 in the presence of p-STAT3 was notably higher than that in the IgG control sample (Fig. 5M).

These findings collectively suggest that the deletion of *CEBPE* induces a feedback loop within the LTF/JAK2/STAT3 regulatory circuitry, driving the ECM degradation and calcification of EPCs.

### 3.6 Constructed cartilage-targeting lipid nanoparticle for delivering *CEBPE* plasmid to CEP tissue

Efficient in vivo methods for the targeted delivery of specific regulatory factors to cells are essential for advancing disease-modifying treatments and clinical applications [31]. Currently, numerous gene editing techniques utilize viral vectors to deliver site-specific targeting nucleases into cells. However, the construction and production of viral vectors are highly cumbersome [32], their cargo capacity is restricted, and they frequently elicit immunogenic responses [33]. In contrast, lipid nanoparticles exhibit excellent biocompatibility and low toxicity, and are capable of delivering nucleic acids into mammalian cells to elicit therapeutic outcomes. Plasmid DNA encoding a protein, suitable for mammalian cell expression, can be encapsulated within a lipid nanoparticle and efficiently delivered into mammalian cells via lipid-mediated transfection, resulting in the expression of a functional protein within the cell [25,34]. To facilitate the specific targeting of *CEBPE* on chondrocytes, a cationic liposome with cartilage-targeting capabilities was designed to deliver the *CEBPE* plasmid to the endplate cartilage (Fig. 6A). TEM analysis revealed that the plasmid-loaded cationic liposomes, Lipo@CEBPE and CAP-Lipo@CEBPE, exhibited particle average sizes of approximately 104.5 nm and 126.9 nm, respectively (Fig. 6B and C). And the surface modification had no significant influence on encapsulation efficiency of CAP-Lipo@CEBPE (Supplementary Fig. 4). Confirming the successful insertion of CAP onto the Liposome surface was further validated by synthesizing CAP-Lipo treated with Nile Red dye and observing the co-localization under a confocal microscope (Fig. 6D). The zeta potential of Lipo@CEBPE is approximately 36.9 mV, whereas that of CAP-Lipo@CEBPE is around 4.7 mV (Fig. 6E). This difference indicates a slight positive charge that may enhance targeting capabilities. The moderate positive charges on the surface of these particles could facilitate adhesion to the negatively charged surfaces of chondrocytes and glycosaminoglycan chains present within cartilage tissue [35]. Stability tests conducted by DLS after one week of incubation in PBS indicated no significant changes in the particle size of CAP-Lipo (Fig. 6F). CAP-Lipo@CEBPE demonstrated efficient transfection of chondrocytes, resulting in high CEBPE expression levels (Fig. 6G and H). To assess the in vivo duration and targeting efficiency of CAP-Lipo@CEBPE, fluorescence imaging was performed on rats at 0, 24, 72, and 96 h following tail vein injections. The fluorescence intensity was observed to gradually increase over time, while it decreased from 72h to 96h, indicating a reduction in the accumulation of CAP-Lipo@CEBPE by 96h (Fig. 6I). Histological analysis of intervertebral disc tissue at the peak time point confirmed the delivery of *CEBPE* plasmid (pEX-6 vector) to CEP, as visualized by fluorescence in frozen tissue sections (Fig. 6J). In clinical applications, liposomes are recognized as a well-established delivery system. Additionally, examination of serum ELISA inflammatory markers (IL-6 and TNF) and H&E-stained sections (major organs—the heart, liver, spleen, lung, and kidney) revealed no significant systemic inflammatory responses, pathological changes, or injuries in model rats following various treatments (Supplementary Fig. 5). These findings indicate the successful construction of a cationic liposome system with cartilage-targeting functionality, capable of efficiently delivering the *CEBPE* plasmid to CEP and promoting increased *CEBPE* expression.

**Fig. 6:**
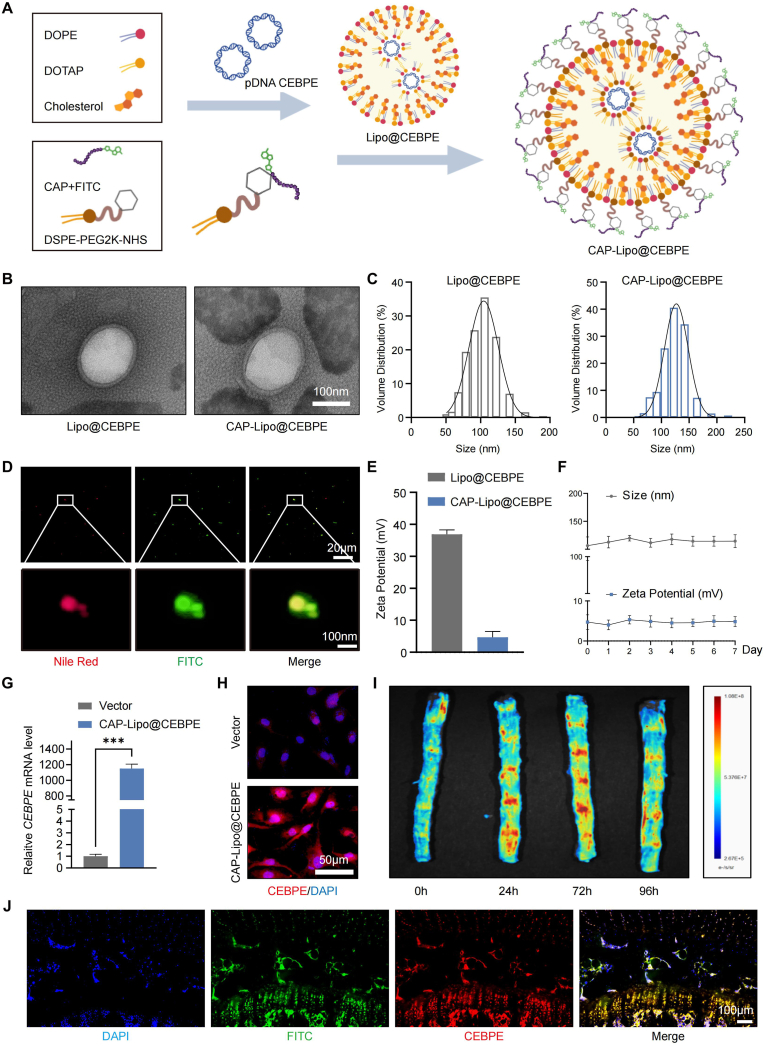
**CAP-Lipo@CEBPE targeted delivery of *CEBPE* to CEP.** (A) Scheme of formulation of CAP-Lipo@CEBPE. (B) TEM images of Lipo@CEBPE and CAP-Lipo@CEBPE (Scale bar = 100 nm). (C) The size distribution of Lipo@CEBPE and CAP-Lipo@CEBPE. (D) Fluorescent colocalization of Lipo (Nile Red) and CAP (FITC) (Scale bar = 20 μm, 100 nm). (E) The surface zeta potential of Lipo@CEBPE and CAP-Lipo@CEBPE. (F) The particle size stability of Lipo@CEBPE and CAP-Lipo@CEBPE. (G) RT-qPCR analysis of the relative levels of *CEBPE* mRNA transcripts in EPCs that had been transfected with Vector or with CAP-Lipo@CEBPE. (H) Representative IF staining of CEBPE in EPCs that had been transfected with Vector or with CAP-Lipo@CEBPE (Scale bar = 50 μm). (I) Fluorescence imaging with IVIS Spectrum imaging system of rat tail after tail intravenous injection with CAP-Lipo@CEBPE for 0h, 24h, 72h, and 96h. (J) Representative confocal images of section of the CEP of rat tail after tail intravenous injection with CAP-Lipo@CEBPE for 72h (Scale bar = 100 μm). ∗∗∗P < 0.001. (For interpretation of the references to colour in this figure legend, the reader is referred to the Web version of this article.)

### 3.7 CAP-Lipo@CEBPE efficaciously modulates degeneration of the rat cartilage endplate

**Fig. 7:**
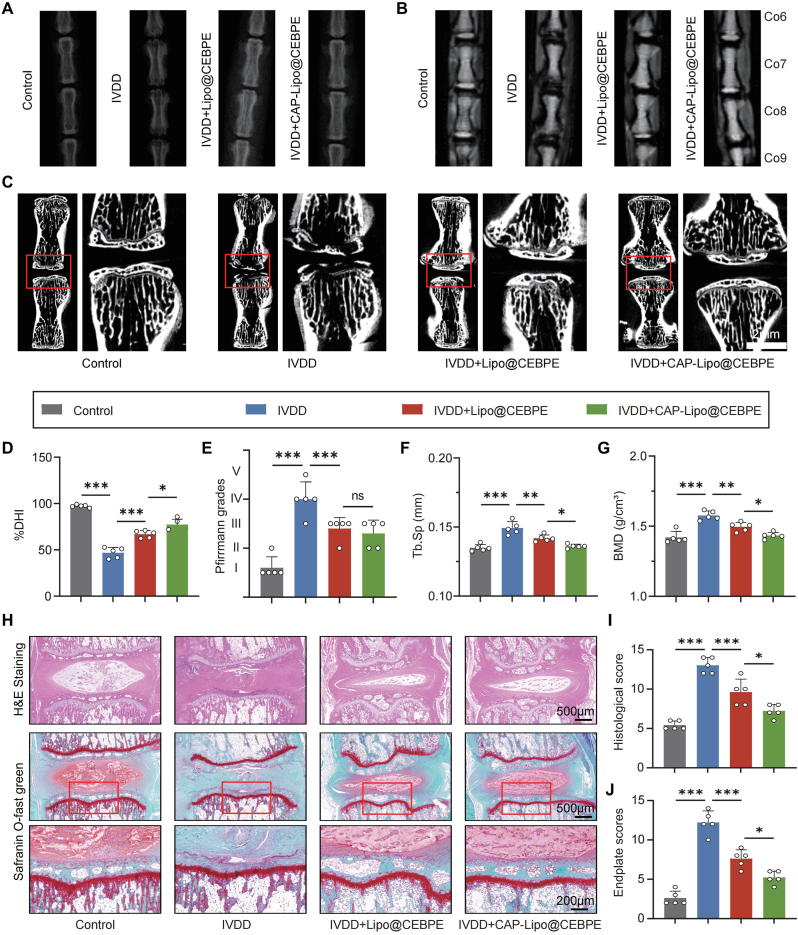
**CAP-Lipo@CEBPE effectively alleviate IVDD progression in vivo.** (A, B) Representative images showing in vivo imaging of coccygeal IVDs in rats immediately after treatment with or without the intravenous injection of Lipo@CEBPE or CAP-Lipo@CEBPE, and representative MRI images of rat coccygeal IVDs treated with the intravenous injection of Lipo@CEBPE or CAP-Lipo@CEBPE (n = 5). (C) μCT images of the coronal plane of rat tail endplates after treatment with or without the intravenous injection of Lipo@CEBPE or CAP-Lipo@CEBPE (Scale bar = 2 mm). (D) DHI (n = 5) of rat coccygeal IVDs. (E) Pfirrmann degenerative grades (n = 5) of rat coccygeal IVDs. (F, G) Quantitative analysis of the Tb. Sp and BMD (n = 5). (H) H&E staining and Safranin O-fast green staining of rat coccygeal IVDs (Scale bar = 500 μm, 200 μm). (I, J) Histological score and Endplate score (n = 5) of rat coccygeal IVDs. n.s. not significant, ∗P < 0.05, ∗∗P < 0.01, and ∗∗∗P < 0.001.

**Fig. 8:**
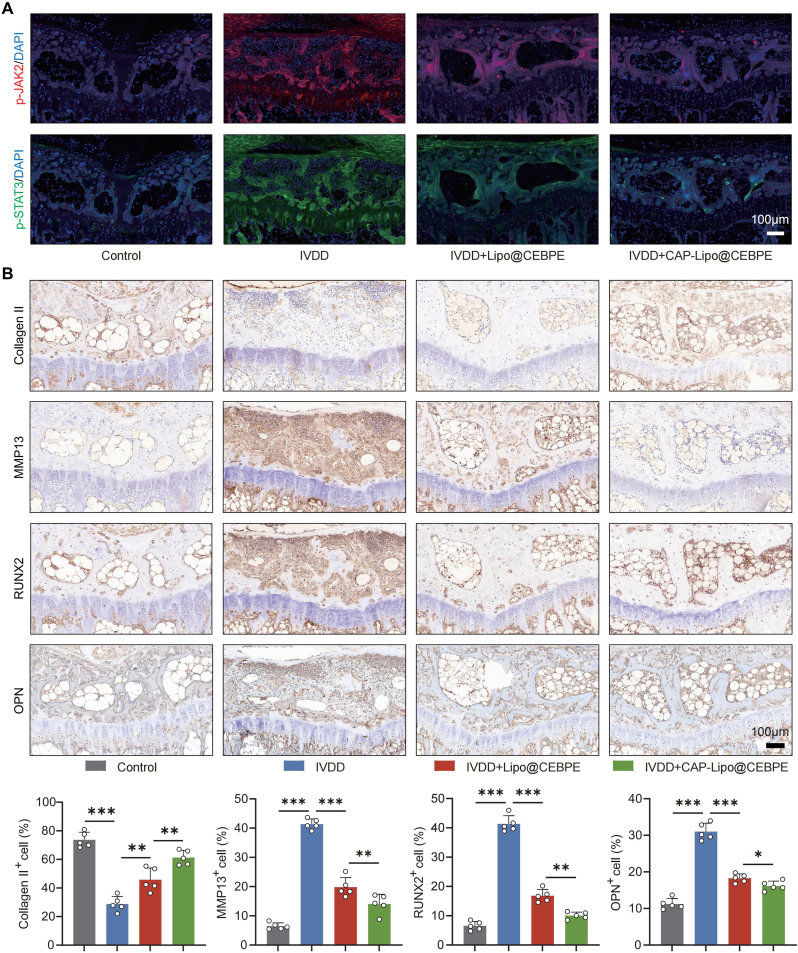
**CAP-Lipo@CEBPE decelerate the ECM degradation and calcification of CEP by modulating JAK2/STAT3 signaling.** (A) Representative IF staining of p-JAK2 (red) p-STAT3 (green) in CEP tissues from rats treated with or without the intravenous injection of Lipo@CEBPE or CAP-Lipo@CEBPE (Scale bar = 100 μm). (B) IHC staining and quantification of Collagen II, MMP13, RUNX2, and OPN expression in CEP tissues (Scale bar = 100 μm). ∗P < 0.05, ∗∗P < 0.01, and ∗∗∗P < 0.001. (For interpretation of the references to colour in this figure legend, the reader is referred to the Web version of this article.)

An IVDD model was constructed to investigate and validate the in vivo reparative effects of CAP-Lipo@CEBPE in IVDD. Imaging analysis revealed that treatments with both Lipo@CEBPE and CAP-Lipo@CEBPE significantly alleviated the reduction in disc height, subchondral bone damage, and loss of nucleus pulposus water content (Fig. 7A, B, D, and E). Notably, CAP-Lipo@CEBPE exhibited a more pronounced therapeutic effect compared to Lipo@CEBPE. μCT analysis indicated that CAP-Lipo@CEBPE mitigated the degeneration-induced increases in trabecular separation (Tb.Sp) and bone mineral density (BMD) in CEP (Fig. 7C–F, and G). Histological staining further confirmed that CAP-Lipo@CEBPE effectively impeded IVDD progression (Fig. 7H–J). IF staining demonstrated a significant downregulation of p-JAK2 and p-STAT3 expression, indicating suppression of the JAK2/STAT3 signaling pathway in CEP tissue following CAP-Lipo@CEBPE treatment (Fig. 8A). IHC staining revealed an increase in Collagen II expression and a reduction in MMP13 expression, indicative of alleviated ECM catabolism. And the expression of CEBPE was rescued following CAP-Lipo@CEBPE treatment (Supplementary Fig. 6). Furthermore, the decreased expression of RUNX2 and OPN in the CEP tissue (Fig. 8B) suggested alleviated calcification of the CEP. These results suggest that *CEBPE*-overexpressing plasmids delivered via CAP-Lipo effectively alleviate EPCs degeneration and ameliorate the impact of IVDD by suppressing the JAK2/STAT3 signaling pathway.

### 3.8 Transcriptomic analysis of differentially expressed mRNAs unveils the therapeutic mechanism for CEP degeneration

**Fig. 9:**
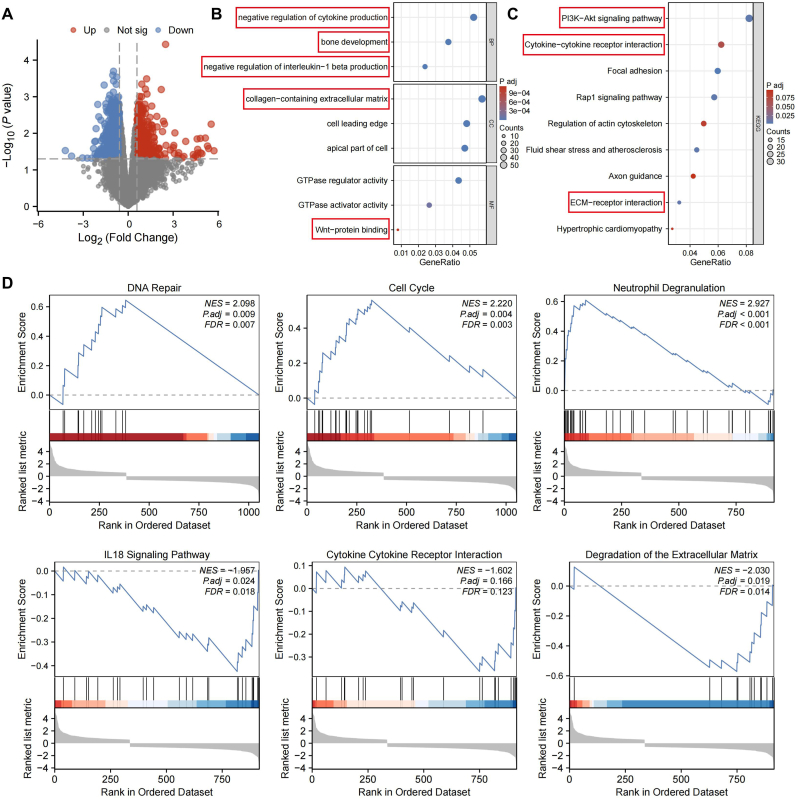
**CAP-Lipo@CEBPE effectively alleviate IVDD progression in vivo.** (A) The volcano plot visualizes the differentially expressed genes between the CAP-Lipo@CEBPE treatment and no treatment groups (red indicates genes that are highly expressed in the treatment group, blue represents genes with low expression levels in the treatment group, and gray signifies genes that show no significant difference). (B) Perform GO enrichment analysis on differentially expressed genes. (C) Perform KEGG enrichment analysis on differentially expressed genes. (D) GSEA enrichment analysis of differential genes. (For interpretation of the references to colour in this figure legend, the reader is referred to the Web version of this article.)

Transcriptomic analysis was conducted to uncover the underlying mechanisms by which CAP-Lipo@CEBPE influences ECM metabolism and cartilage calcification. DEGs between the CAP-Lipo@CEBPE treatment and no treatment groups were visualized using a volcano plot (Fig. 9A). A total of 1062 DEGs were detected, comprising 389 upregulated genes and 673 downregulated genes within the CAP-Lipo@CEBPE-treated group. To gain deeper insights into the underlying mechanisms, we conducted Gene Ontology (GO) and the Kyoto Encyclopedia of Genes and Genomes (KEGG) enrichment analyses. (Fig. 9B and C). The GO analysis revealed strong associations between the differential genes and processes such as negative regulation of interleukin−1 beta production, collagen−containing extracellular matrix, bone developmen. The KEGG analysis revealed significant enrichment in various pathways, notably including cytokine-receptor interactions, PI3K-Akt signaling cascades, and ECM-receptor interactions. Furthermore, GSEA highlighted several differentially enriched pathways, including DNA repair, Cell Cycle, Neutrophil Degranulation, IL18 Signaling Pathway, Cytokine Receptor Interaction and Degradation of the Extracellular Matrix (Fig. 9D). These findings offer a comprehensive understanding of the therapeutic effect of CAP-Lipo@CEBPE, suggesting its role in modulating inflammation, ECM remodeling and calcification through key molecular pathways.

## 4 Discussion

Recent research has highlighted the important role of the CEP in maintaining IVD homeostasis, with CEP dysfunction being closely associated with the development of IVDD [11,36,37]. Understanding the exact function of the CEP in maintaining physiological disc homeostasis and contributing to the pathogenesis of IVDD could offer novel insights into potential therapeutic targets and approaches for the clinical management of IVDD. Thanks to advancements in scRNA-seq technology, several studies have generated comprehensive transcriptomic profiles of IVD tissue at the single-cell resolution. These studies have revealed the identification of specific cell subsets and transcription factors, which could inform the development of targeted therapeutic strategies that focus on these cells and their regulatory pathway [38,39]. In the current study, a significant reduction in the expression of the transcription factor *CEBPE* was observed in tissues affected by CEP degeneration. The functional role of *CEBPE* in CEP degeneration was confirmed through a combination of bioinformatics analysis and experimental validation. Mechanistically, CEBPE was found to interact with the promoter region of *LTF*, modulating its transcriptional activity and consequently activating the JAK2/STAT3 inflammatory signaling cascade. Furthermore, STAT3, acting as a transcription factor for *CEBPE*, regulated its expression and established a positive feedback loop, thereby positioning *CEBPE* as a central molecule in the regulation of CEP degeneration.

*CEBPE*, a member of the CEBP transcription factor family, comprises six distinct proteins: C/EBP-α, β, γ, δ, *ε*, and ζ. The initial CEBP protein was discovered in Steve McKnight's laboratory as a heat-stable nuclear factor present in rat livers. This protein was capable of binding to the CCAAT box motif and specific "core homology" sequences found in certain viral enhancers [40]. By 1992, the other five CEBP family members were discovered, all sharing a conserved bZIP domain at their C-termini [[41], [42], [43], [44], [45], [46], [47], [48]]. The CEBP family plays an essential role in various cellular processes, including the regulation of cell growth and differentiation, immune and inflammatory responses, and the pathogenesis of numerous diseases [17]. Recent studies suggest that the absence of *CEBPE* can influence the expression of genes related to B-cell development (*IL7R* [49]), apoptosis inhibition (*BCL2* [50]), methotrexate resistance (*RASSF4* [51]), and cell survival (*PRAME* [52]), indicating the potential significance of *CEBPE* in disease progression. However, the exact function of *CEBPE* in the pathogenesis of IVDD remains poorly understood.

In this study, a reduction in *CEBPE* expression was observed in degenerated CEP tissue and its expression levels wer correlated with the degree of EPCs damage. To identify the molecular mechanisms by which *CEBPE* deficiency influences CEP homeostasis, knockdown experiments were conducted in chondrocytes to investigate the functional role of *CEBPE*. The results revealed an upregulation of inflammatory cytokines IL-1β and TNF, accompanied by notable inflammatory changes within the cells. Previous studies have indicated that inflammatory cytokines negatively impact ECM synthesis during CEP degeneration [53,54]. This study corroborated these findings, demonstrating that reduced *CEBPE* expression in chondrocytes led to diminished ECM synthesis and enhanced degradation alongside elevated inflammatory cytokine levels. Drawing from studies by Yang et al. and Zhou et al. [55,56], which reported that hypertrophic chondrocytes undergo apoptosis and transdifferentiation into osteoblasts and osteocytes during endochondral ossification, it was hypothesized that CEP degeneration shares similarities with this process. Consequently, chondrocyte calcification was examined, revealing that *CEBPE* deficiency promoted chondrocyte calcification and facilitated the progression toward osteoblast differentiation. Notably, under inflammatory conditions, overexpression of *CEBPE* effectively reversed the reduction in ECM synthesis, as well as the increased degradation and calcification induced by inflammatory cytokines.

*CEBPE*, as a crucial transcription factor, was thoroughly investigated using ChIP-seq to identify potential target genes transcriptionally regulated by it in chondrocytes. This analysis was further supported by the evaluation of RNA-seq data from previous studies, alongside the TRRUST online repository dedicated to transcription factor analysis, which led to the identification of *LTF* as the primary target gene. *LTF*, a member of the transferrin family of iron-binding proteins, is derived through the acidic, pepsin-mediated hydrolysis of the N-terminal segment of lactoferrin obtained from milk [57,58]. Numerous in vitro studies have documented the anti-inflammatory, antiviral, antibacterial, antioxidant, and anticancer properties of *LTF* [59,60]. Additionally, due to its antagonistic effects on cellular activities mediated by IL-1 and LPS, *LTF* demonstrates significant anti-inflammatory, anti-catabolic, and antioxidant actions in the intervertebral disc cells of cattle, mice, and rabbits [61]. This study presents, for the first time, evidence that *LTF*, under the transcriptional regulation of *CEBPE* in chondrocytes, plays a key role in promoting anti-inflammatory responses, inhibiting ECM degradation, and preventing chondrocyte calcification.

JAK2/STAT3 was initially identified as a key component of immune responses and cellular development. However, its diverse roles in inflammation, cellular viability, and gene expression regulation have established it as a critical mediator within the intricate network associated with neurodegenerative processes [62,63]. Zhang et al. demonstrated that 17-AAG alleviates IVDD by inhibiting the JAK2/STAT3 pathway, thus reducing inflammation and ECM degradation in nucleus pulposus cells [64]. Previous research has also shown that *LTF* influences CEP degeneration via the JAK2/STAT3 signaling cascade [30]. In the current study, a more in-depth investigation into the relationship between *CEBPE* and the JAK2/STAT3 pathway was conducted. The findings suggest that the downregulation of *CEBPE* activates the JAK2/STAT3 signaling pathway, which results in reduced ECM synthesis, enhanced ECM degradation, and increased calcification in chondrocytes. Notably, *STAT3* is a multifunctional transcription factor involved in various cellular processes, including inflammation, cellular proliferation, and apoptosis [65]. Upon translocation to the nucleus, *STAT3* not only stimulates the secretion of inflammatory cytokines [66] but also binds to the promoter regions of target genes, thus regulating gene expression. Interestingly, STAT3 was found to bind to the promoter sequence of *CEBPE*, suggesting a potential regulatory interaction between the two factors. Binding site experiments further validated that STAT3 bound to the *CEBPE* promoter, thereby regulating its transcriptional expression. Previous research has shown that the activation of STAT3 in chondrocytes significantly contributes to cartilage degradation and osteophyte formation in osteoarthritis (OA) [67]. As OA progresses, M1 macrophages exacerbate chondrocyte damage and synovial inflammation through the release of pro-inflammatory cytokines such as IL-1β, TNF, and IL-6 [68]. Additionally, phosphorylated STAT3 aids in the maintenance of M1 macrophages, resulting in the secretion of elevated levels of IL-1β and TNF into the cell supernatant [69]. In vivo, a chondrocyte-targeting Lipid nanoparticle (CAP-Lipo@CEBPE) encapsulating an overexpressed *CEBPE* plasmid was formulated. Following tail vein injection, targeted delivery of the overexpressed *CEBPE* plasmid to the CEP significantly delayed IVDD. This was confirmed by the restoration of disc height, inhibition of cartilage ossification, alleviation of tissue morphological damage, and suppression of p-JAK2 and p-STAT3 expression, which are linked to cartilage ECM proteins, matrix degradation, and the JAK3/STAT3 signaling pathway. To further investigate the underlying mechanisms, a transcriptomic analysis was performed. Data from volcano plots, GO and KEGG analyses indicated that CAP-Lipo@CEBPE primarily regulates inflammatory cytokines, ECM remodeling and calcification. Moreover, GSEA revealed that pathways related to DNA repair, the cell cycle, and the Cytokine Receptor Interaction may also be involved in this process.

In conclusion, the research identifies a positive feedback loop within the degenerative process of CEP. As CEP undergoes degeneration, inflammatory responses are activated, triggering the release of cytokines such as IL-1β and TNF. These cytokines then induce a reduction in the expression of *CEBPE*, which in turn influences the transcriptional activity of *LTF*. This leads to the activation of the JAK2/STAT3 signaling cascade, increasing phosphorylated STAT3 levels. The p-STAT3 molecules bound to the *CEBPE* promoter region, suppressing its transcriptional expression. This suppression further exacerbates the inflammatory response, prompting the release of additional inflammatory cytokines, thus accelerating the degenerative process of CEP and ultimately leading to IVDD. Notably, the administration of an *CEBPE*-overexpressing plasmid delivered by chondrocyte-targeting Lipid nanoparticle efficiently alleviated the ECM degradation and calcification process of CEP and substantially mitigated IVDD progression.

## CRediT authorship contribution statement

**Jiangminghao Zhao:** Writing – original draft, Visualization, Software, Formal analysis, Validation, Project administration. **Peichuan Xu:** Writing – review & editing, Methodology, Software, Formal analysis. **Tao Li:** Writing – review & editing, Validation. **Jinghong Yuan:** Software, Validation, Resources. **Wenrui Zhao:** Data curation, Software. **Rui Ding:** Data curation, Methodology. **Guoyu Yang:** Supervision, Validation. **Jiajun Xie:** Validation. **Guanfeng Huang:** Software. **Zhidong Peng:** Methodology. **Xinxin Miao:** Visualization, Project administration. **Xigao Cheng:** Writing – review & editing, Investigation, Visualization, Conceptualization.

## Ethics statement

This study was approved by the Ethics Committee of Nanchang University (Approval No.NCULAE-20221031075) and (Approval No. Review (2023) No. (06)).

## Funding

This study was supported by the National Science Founding of China (No. 82060403) and International Scientific and Technological Cooperation Project (No. 20232BBH80001).

## Declaration of competing interest

The authors declare that they have no known competing financial interests or personal relationships that could have appeared to influence the work reported in this paper.

## Figures and Tables

**figs1:**
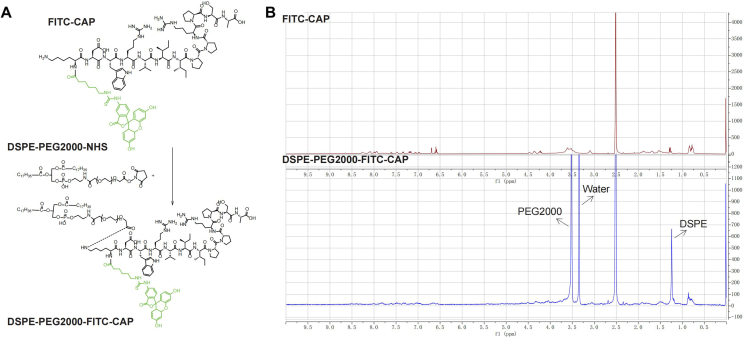
**The synthesis and detection of DSPE-PEG2000-FITC-CAP.** (A) Composite route of DSPE-PEG2000-FITC-CAP. (B) NMR spectroscopy report.

**figs2:**
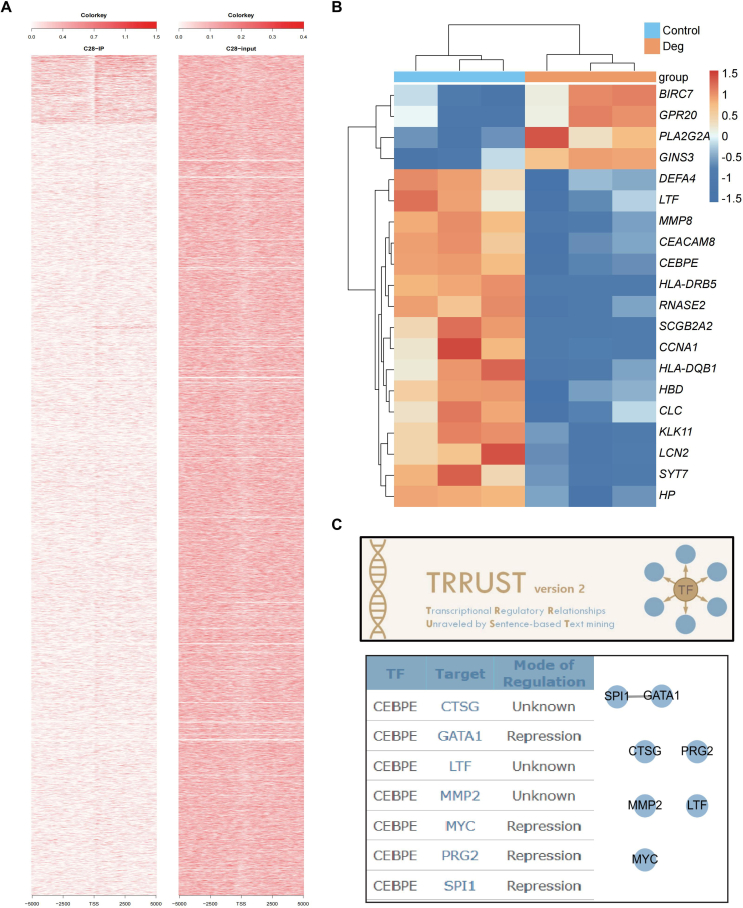
**Screening for target genes of *CEBPE* in the cartilaginous endplate.** (A) ChIP-seq heatmap. (B) Heatmap of the top 20 DEGs. (C) Target gene prediction using the TRRUST transcription factor database.

**figs3:**
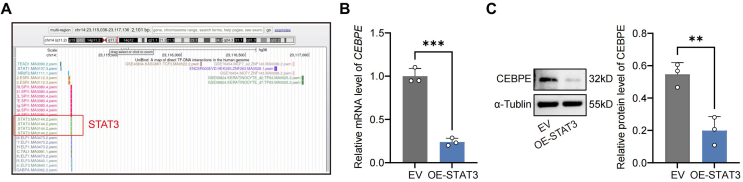
**STAT3, as a transcription factor of *CEBPE*, inhibits the expression of *CEBPE*.** (A) Analysis of transcription factors in the *CEBPE* promoter region using the UCSC Genome Browser. (B) RT-qPCR analysis of the relative levels of *CEBPE* mRNA transcripts in EPCs that had been transduced with *STAT3*-overexpressing plasmids (OE-STAT3), or with EV. (C) WB analysis of CEBPE expression in EPCs that had been transduced with OE-STAT3, or with EV. ∗∗P < 0.01, and ∗∗∗P < 0.001.

**figs4:**
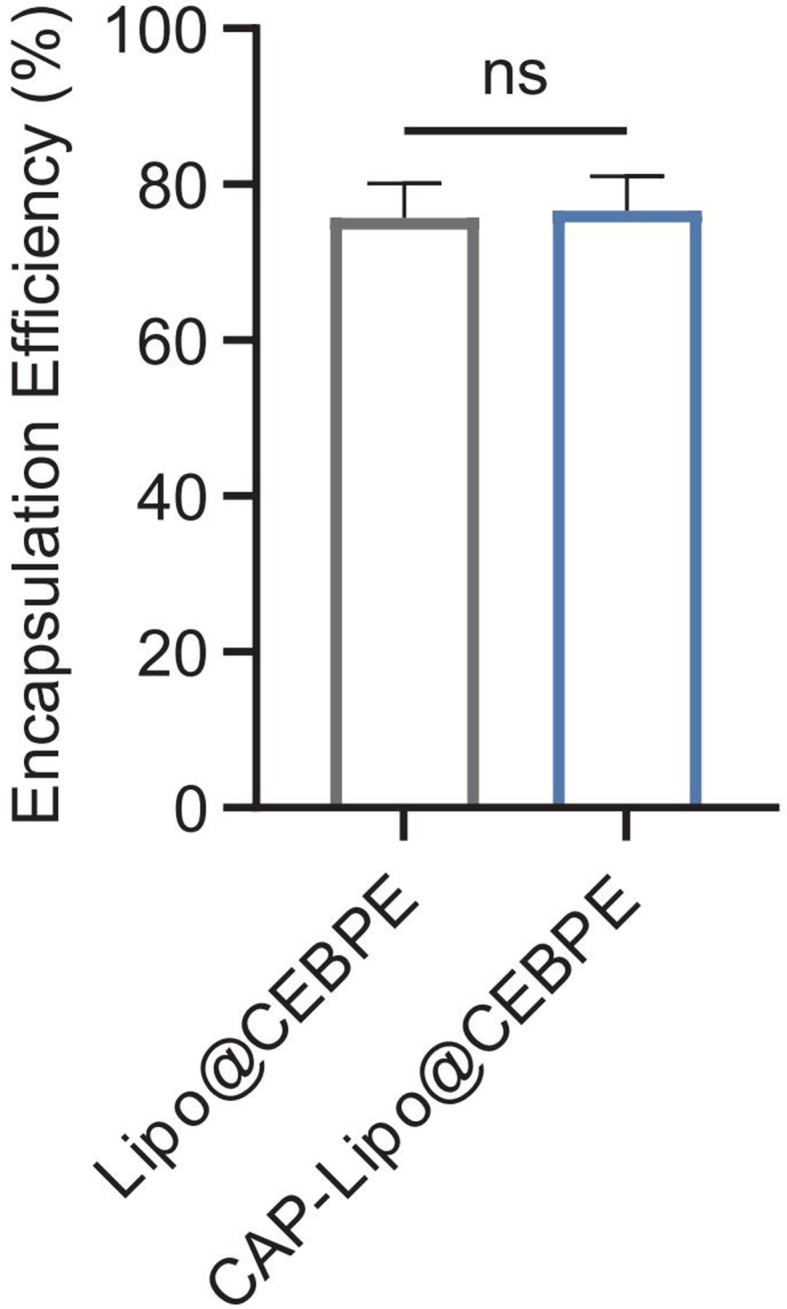
The encapsulation efficiency of Lipo@CEBPE and CAP-Lipo@CEBPE.

**figs5:**
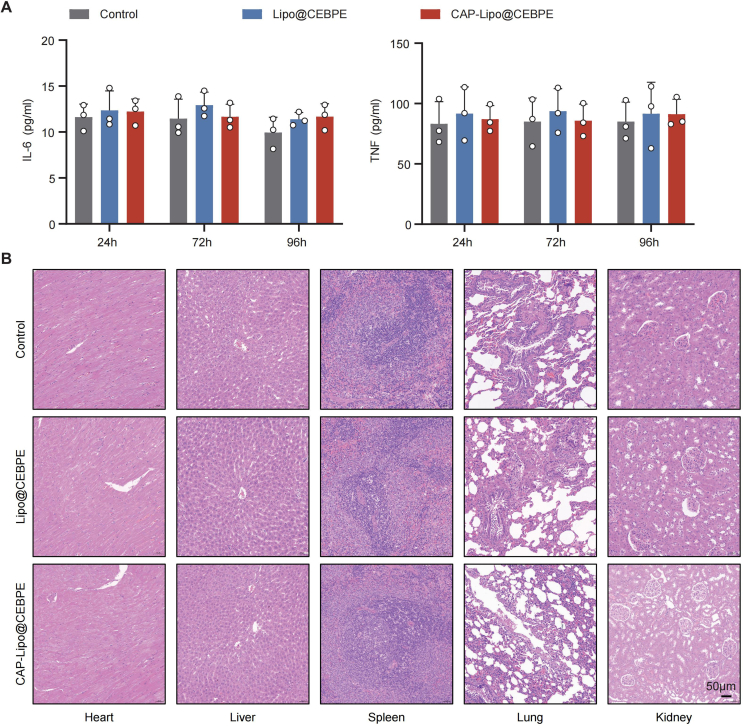
**Safety evaluations of normal rats with different treatments.** (A) IL-6 and TNF in serum measured by ELISA. (B) Representative images of H&E staining of major organs from rats after different treatments (Scale bar = 50 μm).

**figs6:**
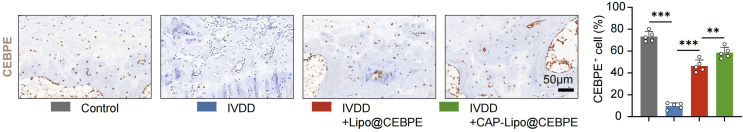
The expression of CEBPE following CAP-Lipo@CEBPE treatment in CEP.

## Data Availability

Data will be made available on request.

## Appendix A Supplementary data

Multimedia component 1 Demographic data of intervertebral disc donors. Multimedia component 1

Multimedia component 2 The primer sequences. Multimedia component 2
